# Dose‐Dependent Effect of Crocin on Depressive and Anxiety Symptom Scores: A GRADE‐Assessed Systematic Review and Meta‐Analysis of Randomized Controlled Trials

**DOI:** 10.1155/da/8303882

**Published:** 2026-10-30

**Authors:** Roya Behrouzi, Fatemeh Mehdipoor, Kimia Torabinasab, Hossein Shahinfar

**Affiliations:** ^1^ Department of Nutrition School of Public Health Iran University of Medical Sciences Tehran Iran iums.ac.ir; ^2^ Student Research Committee Faculty of Nutrition and Food Technology Shahid Beheshti University of Medical Sciences Tehran Iran sbmu.ac.ir; ^3^ Nutritional Health Research Center School of Health and Nutrition Lorestan University of Medical Sciences Khorramabad Iran lums.ac.ir

**Keywords:** anxiety, crocin, depression, dose–response, meta-analysis

## Abstract

**Background:**

Although several clinical trials have examined the effects of crocin on the symptoms of depression and anxiety, their findings have not yet been synthesized comprehensively. Therefore, this systematic review and meta‐analysis aimed to determine the dose‐dependent effect of crocin supplementation on depressive and anxiety symptom scores.

**Methods:**

A comprehensive search was performed in the PubMed, Scopus, and ISI Web of Science until April 2026. The change in depressive and anxiety symptom scores for each 30 mg/d increase in crocin supplementation was estimated and then the standardized mean difference (SMD) with 95% CI was calculated. We also assessed the publication bias, sensitivity analysis and heterogeneity by standard methods. Certainty of evidences was evaluated through the GRADE Tool.

**Results:**

Fourteen randomized controlled trials (RCTs) were included. Each 30 mg/day increasing in crocin supplementation can decreased scores of depressive symptoms by −3.37 (95% CI: −4.80, −1.94; *I* ^2^ = 97.1%, GRADE = moderate). A slight linear reduction in the depressive symptom scores was observed (*p*_nonlinearity_ = 0.47, *p*_dose–response_ = 0.001). Each 30 mg/day crocin can led to −2.73 reduction in anxiety symptom scores (95% CI: −4.17, −1.06; *I* ^2^ = 97%, GRADE = high). Anxiety symptom scores reduced with a slight linear slope (*p*_nonlinearity_ = 0.9, *p*_dose–response_ = 0.02).

**Conclusion:**

In conclusion, crocin supplementation may improve symptom scores of both depression and anxiety in a dose–response manner. However, these results are based on clinical trials with relatively small sample sizes, almost conducted in one country. Well‐designed RCTs across diverse regions with adequate sample sizes are required to confirm these findings.

**Trial Registration:** Prospero Code: CRD42024591281

## 1. Introduction

Mental disorders are considered as a main contributor to the disease burden [1]. Depression and anxiety are among the substantially prevalent mental disorders across various demographic and health conditions as evidences by rigorous studies [2, 3] and impose detrimental effects on quality of life [4]. In 2023, depressive disorders accounted for 5.65% of the global burden of disease in terms of years lived with disability (YLDs) (refer to section Nomenclature), highlighting their significant impact on quality of life. Similarly, anxiety disorders contributed 5.6% to the global YLDs, with a disproportionately greater effect observed among women [5]. Notably, depression and anxiety frequently co‐occur, and their high comorbidity has been attributed to overlapping pathophysiological mechanisms, including dysregulation of stress response systems, neuroinflammation, and alterations in monoaminergic neurotransmission [6–8]. People living with mental health disorders may experience a range of distressing psychological and physical manifestations. Symptoms specific to depression commonly include anhedonia, poor concentration, feelings of hopelessness, insomnia or hypersomnia, fatigue, psychomotor agitation, and changes in appetite or weight. Additionally, anxiety disorders are characterized by excessive worry, restlessness, difficulty concentrating, sleep disturbances, and somatic symptoms such as heart palpitations and gastrointestinal discomfort [9, 10]. Among various therapeutic strategies employed for mental issues, pharmacological treatments have shown their effectiveness [10, 11]. In addition, high‐quality studies have evidenced precious effects of cognitive behavioral therapy (CBT) on patients with depressive and anxiety symptoms [11]. Alongside psychological interventions, growing attention has been directed toward nutritional and herbal approaches as complementary strategies for managing symptoms associated with mental disorders. Systematic reviews and meta‐analyses of clinical trials have reported reductions in depression and/or anxiety symptom scores following supplementation with herbal products such as lavender [12], Passiflora [13], St. John’s wort [14], kava extract [15], and chamomile [16]. Moreover, crocin, a carotenoid found predominantly in saffron (*Crocus sativus* L.), has emerged as a promising candidate due to its potential neuroprotective and mood‐enhancing effects, particularly in individuals suffering from depressive disorders. Preclinical studies suggest that crocin may exert antidepressant‐like and anxiolytic‐like effects through multiple mechanisms, including attenuation of oxidative stress and neuroinflammation, inhibition of inflammatory signaling pathways, and enhancement of neurotrophic effects [17–19]. In a 12‐week double‐blinded randomized controlled trial (RCT) conducted on patients diagnosed with depression and anxiety, patients receiving 50 mg/day saffron capsules exhibited a notable reduction in the Beck Depression Inventory (BDI) and Beck Anxiety Inventory (BAI) scores [20]. The effect of crocin as an adjunctive therapy in major depressive disorder (MDD) was explored in a 4‐week RCT, which demonstrated that the administration of 30 mg/day crocin tablets, in conjunction with serotonin reuptake inhibitors (SSRIs), led to a decrease in BDI, BAI, and General Health Questionnaire (GHQ) scores when compared to a placebo [21]. These results were replicated in trials conducted on various populations [22, 23]. Conversely, a separate 12‐week RCT focusing on patients undergoing coronary artery bypass grafting did not find evidence supporting the positive impact of saffron on mental health [24]. Given these controversial results, we opted to conduct a systematic review and meta‐analysis of RCTs clarifying the dose‐dependent effects of crocin on depressive and anxiety symptom scores.

## 2. Methods

We conducted this systematic review and meta‐analysis in accordance with the PRISMA‐2020 guidelines [25] (Table S1). The review protocol was registered on PROSPERO under the registration number CRD42024591281. Following the PICOS framework, our inclusion criteria addressed participants (adults aged 18 and older), the intervention (crocin supplementation) compared to either a placebo or a standard antidepressant, outcomes (change in depressive and anxiety symptom scores), and study design (randomized clinical trials with either parallel or cross‐over formats) [26].

### 2.1. Search Strategy

Roya Behrouzi and Fatemeh Mehdipoor carried out a thorough literature search across PubMed, Scopus, and the ISI Web of Science databases up to April 5, 2026, aiming to identify studies that investigated the impact of crocin supplementation on depressive and anxiety symptom scores. The search strategy combined relevant keywords and medical subject headings (MeSH), including terms like “crocin,” “saffron,” “*crocus sativus*,” “depression,” “anxiety,” “mental health,” “mental disorder,” “randomized controlled trial,” “RCT,” “placebo,” and “trial,” using Boolean operators (AND and OR) to improve search precision. The complete search strategy is detailed in Table S2. In addition to the database search, reference lists of the selected articles and related systematic reviews were manually reviewed. A supplementary search was also performed using Google Scholar to identify any potentially missed studies.

### 2.2. Inclusion and Exclusion Criteria

Study selection was guided by the PICOS framework: (1) participants: adults aged 18 years or older; (2) intervention: crocin supplementation in any dose or form; (3) comparison: placebo or conventional antidepressant treatment; and (4) outcome: the change in depressive and anxiety symptom scores, assessed using validated tools such as BDI, BAI, Hamilton Depression and Anxiety Rating Scale (HAM‐D and HAM‐A), or Depression Anxiety and Stress Scale (DASS). Studies were included only if they used standardized and psychometrically validated instruments to ensure consistency and comparability across trials and (5) study design: RCTs. Studies were excluded if they were not RCTs (e.g., observational studies and case series), lacked a control group, used nonvalidated or ad‐hoc measures, involved animal or in vitro models, did not assess depressive and anxiety symptom scores, or did not provide sufficient data for meta‐analysis (such as missing means or standard deviations [SDs]). Although the initial search did not impose language restrictions, only articles published in English were ultimately considered.

### 2.3. Data Extraction

Two independent reviewers (Roya Behrouzi and Fatemeh Mehdipoor) systematically extracted data using a standardized form designed to capture key study characteristics. Extracted information included study details (first author, publication year, and country), participant demographics (sample size, average age, sex distribution, and baseline mental health status), intervention details (crocin dose, duration of supplementation, and formulation), control group information (description of the placebo), and outcome measures (mean changes in depressive and anxiety symptom scores). Any disagreements between the reviewers were resolved through discussion or, if needed, with input from a third reviewer (Hossein Shahinfar).

### 2.4. Quality Assessment

Each study’s quality was assessed using the revised Cochrane Risk of Bias tool for Randomized Trials (RoB 2) [27]. The assessment covered five key domains: (1) bias arising from the randomization process; (2) bias due to deviations from the intended interventions; (3) bias due to missing outcome data; (4) bias in measuring the outcome; and (5) bias in the selection of the reported results. The judgement of risk of bias was predicated on the responses to each question within each domain and rated as low, high, or some concern. The overall quality rating was then determined as follows: low (if no domains were rated as high risk or some concerns), some concerns (where one or more domains were judged to have some concerns), and high (if at least one domain was rated high or multiple domains were rated some concerns). Any disagreements in the bias assessment were resolved through discussion.

### 2.5. Statistical Analysis

All statistical analyses were performed using Stata version 16. The primary outcome was measured as the standardized mean difference (SMD) in depressive and anxiety symptom scores between the crocin and control groups. For each study, the mean and SD of depressive and anxiety symptom scores at baseline and at the end of the trial for both the crocin and control groups were extracted. We computed the change in each group as the difference between the post‐intervention value and the baseline value (i.e., end of trial minus baseline). Specifically, the treatment effect was calculated as follows: (end of follow‐up_treatment_ − Baseline_treatment_) − (End of follow‐up_control_ − Baseline_control_). These quantities were then used to derive the SMDs for the meta‐analytic synthesis. For studies that did not directly report means and SDs, other available statistical data were converted using appropriate formulas. Specifically, when pre‐ and post‐treatment SDs were provided without the SD of the difference, it was calculated using the formula: SD difference = √[(SD pre‐treatment)^2^ + (SD post‐treatment)^2^ − (2 × R × SD pre‐treatment × SD post‐treatment)], assuming a correlation coefficient (*R*) of 0.8 as a conservative estimate within the 0–1 range [28]. If the standard error of the mean (SEM) was reported instead of SD, it was converted using SD = SEM × √*n*, where “*n*” is the number of participants in the group. For studies reporting medians and interquartile ranges (IQRs) rather than means and SDs, values were estimated assuming a symmetric distribution, using the formula: SD = IQR/1.35 [29]. When outcome data were only available in graphical format, values were extracted using the Get Data Graph Digitizer version 2.24 [30]. Initially, a random effect model was applied, and results were visualized through forest plots. Heterogeneity among studies was assessed using the *I* ^2^ statistic, with values over 50% and a *p*‐value below 0.1 considered to indicate substantial heterogeneity [31]. To explore potential sources of heterogeneity, subgroup analyses were conducted based on variables such as participants’ health status, treatment duration, sex, quality of trials, and outcome assessment tool. Sensitivity analysis was carried out to assess the impact of individual trials on the overall effect size. Heterogeneity and homogeneity were further assessed using the *I* ^2^ and *χ* ^2^ statistics, respectively [32]. To evaluate publication bias, funnel plots were visually inspected. Also, Egger’s regression and Begg’s correlation tests were used to statistically assess asymmetry [33, 34]. Additionally, to estimate the MD in depressive and anxiety symptom scores associated with every 30 mg/day increase in crocin intake, we applied the method developed by Crippa and Orsini [35]. This approach required the dose of crocin (mg/day), the mean and SD of change in depressive and anxiety symptom scores, and the number of participants per group. Finally, a dose–response meta‐analysis was performed to examine the relationship between crocin dosage and depressive and anxiety symptom scores [36]. All statistical tests were two‐tailed, and a *p*‐value less than 0.05 was considered statistically significant.

### 2.6. Grading the Evidence

We assessed the strength of the overall evidence using the GRADE (Grading of Recommendations Assessment, Development, and Evaluation) approach from the following website: http://gradepro.org [37]. Two reviewers (Roya Behrouzi and Hossein Shahinfar) independently rated the quality of evidence based on several criteria, including risk of bias, inconsistency, indirectness, imprecision, and potential publication bias. The evidence could also be downgraded based on the following factors: risk of bias (if up to 20% of included studies rated as high risk of bias), inconsistency (if heterogeneity across studies, indicated by an *I* ^2^ statistic, was higher than 50% and high heterogeneity could not be explained in subgroup analysis), indirectness (when study findings could not be directly generalized to the target population or intervention due to differences in study populations, interventions, or comparators), imprecision (a lack of optimal information size or confidence intervals (CIs) that included the null value), publication bias (potential systematic differences between published and unpublished studies were considered). Conversely, the quality of evidence could be upgraded if there was a strong effect size or a clear dose–response relationship. According to these assessments, each outcome was categorized as having high, moderate, low, or very low certainty. Any disagreements were resolved through discussion until consensus was reached.

## 3. Results

### 3.1. Search Results

The summary of the study selection procedure is shown in Figure S1. Of 1149 records which were identified through initial search in the databases, 325 were duplicated and removed. Then, title and abstracts of remaining papers were screened and 753 were also excluded. In the next step, a full‐text evaluation of 72 remaining records led to the removal of 58 articles as they did not meet the inclusion criteria due to incorrect study design (*n* = 1), non‐English publication (*n* = 1), assessing other saffron constituents (*n* = 25), incorrect outcomes (*n* = 29), and congress abstract (*n* = 2). The detailed exclusion list, including the rationale for each removal, is provided in Table S3. Finally, 14 RCTs met our inclusion criteria. Of the included studies, 13 assessed depressive symptom scores and 12 assessed anxiety symptom scores.

### 3.2. Study Characteristics

The general characteristics of the included studies are summarized in Table 1. These RCTs were published between 2015 and 2024 with a total number of 678 participants. Of these participants, 342 were assigned to the intervention group and 336 to the control group. The median duration of intervention was 8 weeks (range: 4–16 weeks). The mean age of participants in the included studies ranged from 27.93 to 65.56 years. All studies were conducted in Iran except one of them that conducted in Japan [40]. Two studies included only women [45, 47], and three studies included only men [41, 42, 46], but the rest included both men and women. The dose of crocin ranged from 0.6 to 30 mg per day, as detailed in Table 1. A parallel‐study design was used in all trials. The studies included participants with neuropsychiatric disorders (*n* = 6), healthy individuals (*n* = 4), cancer patients (*n* = 2), and individuals with metabolic diseases (*n* = 2). Regarding depression outcomes, the included trials assessed symptoms using the BDI (*n* = 10), HAM‐D (*n* = 1), and DASS (*n* = 2). For anxiety outcomes, the BAI (*n* = 8), HAM‐A (*n* = 2), and DASS (*n* = 2) were used. According to RoB 2 results as presented in Tables 1 and S4, the risk of bias in five studies was graded some concerns, while the rest of them were graded low.

**Table 1:** Characteristic of included studies in the meta‐analysis.

First author (year)	Country	Study design	Health condition	Sex (% female)	Sample size (Int/Con)	Trial duration (week)	Means age	ROB 2	Dose of supplement (mg/day)	Type of supplement (Int/Con)	Outcomes
Talaei et al. 2015 [21]	Iran	Randomized, double‐blind, placebo‐controlled, parallel trial	Major depressive disorder	Both (85)	20/20	4	36.2	Low	30	Crocin tablet + SSRI/Placebo + SSRI	BDI, BAI
Abdimanesh et al. 2017 [38]	Iran	Randomized, double‐blind, placebo‐controlled, parallel trial	Coronary artery disease	Both (52.6)	19/19	8	56.1	Low	30	Crocin tablet/placebo	BDI
Jam et al. 2017 [39]	Iran	Randomized, double‐blind, placebo‐controlled, parallel trial	Metabolic syndrome	Both (66.6)	16/17	8	43.16	Some concerns	30	Crocin tablet/placebo	BDI
Nishide et al. 2018 [40]	Japan	Randomized, double‐blind, placebo‐controlled, parallel trial	Healthy	Both (47.6)	10/11	4	37.7	Low	0.6	Crocin tablet/placebo	DASS‐D, DASS‐A
Ghaderi et al. 2019 [41]	Iran	Randomized, double‐blind, placebo‐controlled, parallel trial	Methadone maintenance participants with depressive and anxiety symptoms	Male (0)	26/27	8	44.5	Low	30	Crocin tablet/placebo	BDI, BAI
Khalatbari‐mohseni et al. 2019 [42]	Iran	Randomized, double‐blind, placebo‐controlled, parallel trial	Methadone maintenance participants	Male (0)	25/25	8	40.1	Low	30	Crocin tablet/placebo	BDI, BAI
Pakfetrat et al. 2019 [43]	Iran	Randomized, double‐blind, placebo‐controlled, parallel trial	Burning mouth syndrome	Both (68)	26/21	11	52.92	Some concerns	30	Crocin tablet/Citalopram	HAM‐D, HAM‐A
Kazemi et al. 2021 [44]	Iran	Randomized, double‐blind, placebo‐controlled, parallel trial	Obsessive compulsive disorder	Both (78)	25/25	4	32.8	Low	15	Crocin tablet/Fluoxetine	HAM‐A
Salek et al. 2021 [45]	Iran	Randomized, double‐blind, placebo‐controlled, parallel trial	Breast cancer	Female (100)	36/36	16	NR	Low	30	Crocin tablet/placebo	BDI, BAI
Jomehpour et al. 2022 [46]	Iran	Randomized, double‐blind, placebo‐controlled, parallel trial	Opioid users in detoxification period	Male (0)	36/35	6	59.2	Some concerns	30	Krocina tablet/placebo	DASS‐21
Kolahdooz et al. 2023 [47]	Iran	Randomized, double‐blind, placebo‐controlled, parallel trial	Postpartum depression	Female (100)	32/32	12	27.93	Some concerns	15	Crocin tablet/Sertraline	BDI‐II, BAI‐II
Ebrahimi et al. 2024 [48]	Iran	Randomized, double‐blind, placebo‐controlled, parallel trial	Carcinoma	Both (59)	23/23	4	65.56	Low	30	Crocin tablet/placebo	BDI, BAI
Kouchaki et al. 2024 [49]	Iran	Randomized, double‐blind, placebo‐controlled, parallel trial	Multiple sclerosis	Both (71.11)	25/20	8	36	Some concerns	30	Crocin tablet/placebo	BDI, BAI
Mahdavi et al. 2024 [50]	Iran	Randomized, double‐blind, placebo‐controlled, parallel trial	Cigarette smokers	Both (18.75)	23/25	12	34.39	Low	30	Crocin tablet/placebo	BDI, BAI

Abbreviations: BAI, Beck Anxiety Inventory; BDI, Beck Depression Inventory; DAAS‐A, Depression Anxiety and Stress Scale‐Anxiety; DAAS‐D, Depression Anxiety and Stress Scale‐Depression; HAM‐A, Hamilton Scale of Anxiety; HAM‐D, Hamilton Scale of Depression; SSRI, selective serotonin reuptake inhibitor.

### 3.3. The Effect of Crocin on Depressive Symptom Scores

Linear dose–response meta‐analysis revealed that each 30 mg/day increase in crocin supplementation reduced depressive symptom scores by 3.37 (95% CI: −4.80, −1.94; *I* ^2^ = 97.1%) (Figure 1). Furthermore, nonlinear dose–response relationship between crocin and depressive symptom scores is demonstrated in Figure 2. and Table 2. Depressive symptom scores reduced with a linear and slight slope (*p*_non-linearity_ = 0.47 and *p*_dose–response_ = 0.001) up to 30 mg/day crocin supplementation.

**Figure 1:**
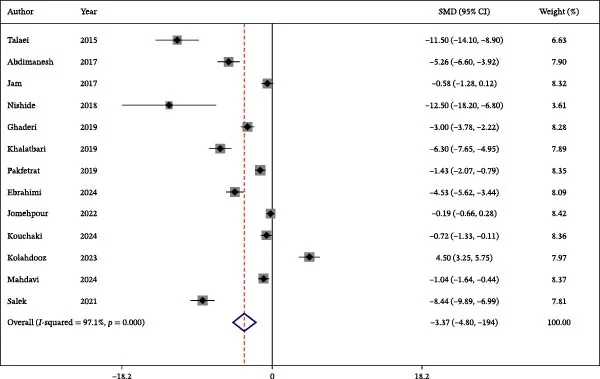
Linear dose–response relations between crocin and standardized mean difference in depressive symptom scores. Weights are from random effects analysis.

**Figure 2:**
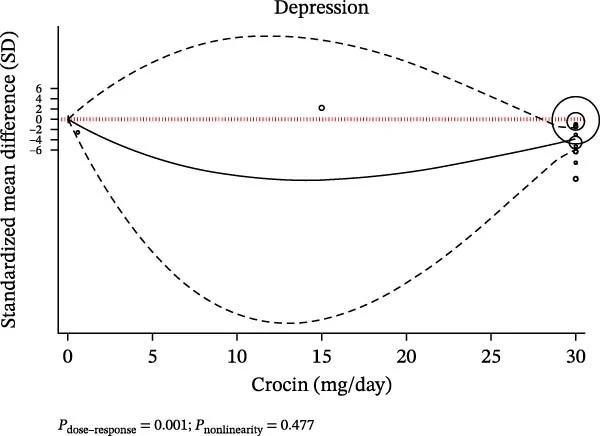
Nonlinear dose–response relations between crocin and standardized mean difference in depressive symptom scores.

**Table 2:** The effect of different doses of crocin on depressive and anxiety symptom scores from the nonlinear dose–response meta‐analysis (standardized mean differences and 95% confidence interval).

Crocin (mg/day)	0 (Ref)	5	10	15	20	25	30	35	40	45	50
Depressive symptoms	0	−7.32 (−25.77, 11.13)	−11 (−37.84, 15.84)	−11.75 (−38.93, 15.43)	−10.31 (−31.80, 11.18)	−7.41 (−19.25, 4.42)	−3.79 (−5.89, −1.68)	−0.04 (−12.43, 12.36)	3.71 (−20.69, 28.12)	7.46 (−29.01, 43.93)	11.21 (−37.34, 59.76)
Anxiety symptoms	0	−1.02 (−7.66, 5.61)	−1.84 (−11.47, 7.80)	−2.48 (−12.23, 7.28)	−2.99 (−10.75, 4.77)	−3.41 (−7.99, 1.16)	−3.80 (−6.55, −1.04)	−4.17 (−9.90, 1.55)	−4.55 (−14.43, 5.33)	−4.93 (−19.14, 9.28)	−5.30 (−23.90, 13.29)

Visual inspection of funnel plot (Figure S2) indicated an asymmetry in the effect of crocin on depressive symptom scores. This finding was confirmed by Egger’s linear regression (*p* = 0.008) and Begg’s rank correlation (*p* = 0.01). Results remained stable after excluding each study from the analysis (MD range: −4.80, −1.94).

### 3.4. The Effect of Crocin on Anxiety Symptom Scores

The dose–response analysis of crocin supplementation on anxiety symptom scores showed a significant linear association. Each 30 mg/day increment in crocin led to a 2.73 reduction in anxiety symptom scores (95% CI, −4.17, −1.28; *I* ^2^ = 97%) (Figure 3). In addition, the dose–response analysis of crocin supplementation revealed a slight linear reduction for anxiety symptom scores in doses of 30 mg/day (*p*_non-linearity_ = 0.9 and *p*_dose–response_ = 0.02) (Figure 4 and Table 2).

**Figure 3:**
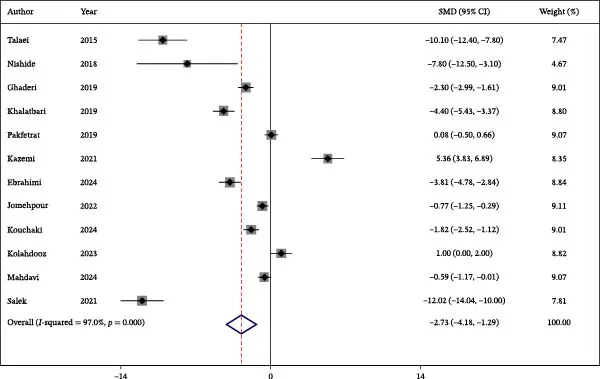
Linear dose–response relations between crocin and standardized mean difference in anxiety symptom scores. Weights are from random effects analysis.

**Figure 4:**
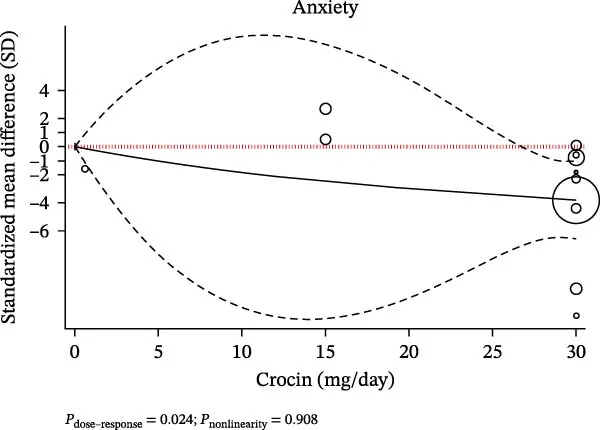
Nonlinear dose–response relations between crocin and standardized mean difference in anxiety symptom scores.

Based on visual inspection of funnel plot (Figure S3) as well as Egger’s (*p* = 0.12) and Begg’s tests (*p* = 0.15), no significant publication bias was addressed. Results remained stable after excluding each study from analysis (MD range: −4.17, −1.28).

### 3.5. Subgroup Analysis

Subgroup analysis could not reduce high levels of heterogeneity for both depressive and anxiety symptom scores (Table 3). In our analysis, the subgroup of neuropsychiatric disorders included participants with the following medical conditions: MDD, multiple sclerosis patients, postpartum depression, burning mouth syndrome, patients under methadone maintenance treatment with depressive and anxiety symptoms, and participants with obsessive–compulsive disorder. The metabolic diseases subgroup comprised studies on: metabolic syndrome and coronary artery disease. The cancer subgroup included esophageal carcinoma and breast cancer patients. Participants in the remaining studies, who did not present clinical conditions, were classified in the healthy population. When studies were stratified based on the health status of participants, sex, duration of intervention, and trial quality, the results remained significant for depressive symptom scores. However, stratifying outcome assessment tool showed nonsignificant result for DASS‐21 (ES: −0.27; 95% CI: −0.73, 0.19). Other categories of assessment tools, such as BDI (ES: −1.93; 95% CI: −2.21, −1.64) and HAM‐D (ES: −1.43; 95% CI: −2.07, 0.78), were significant. Moreover, a significant reduction was seen across all subgroups for anxiety symptom scores.

**Table 3:** Subgroup analysis of included trials in meta‐analysis of the effect of crocin on depressive and anxiety symptoms.

Group	Number	SMD (95% CI)	*p*‐Value	*I* ^2^ (%)	*p*‐Heterogeneity	*p* For between subgroup heterogeneity
Depressive symptom scores						
Health status	—	—	—	—	—	<0.001
Neuropsychiatric disorders	5	−1.20 (−1.57, −0.84)	<0.001	97.5	<0.001	—
Metabolic diseases	2	−1.57 (−2.19, −0.95)	<0.001	97.3	<0.001	—
Healthy	4	−0.95 (−1.30, −0.59)	<0.001	96.5	<0.001	—
Cancer	2	−5.93 (−6.81, −5.06)	<0.001	94.4	<0.001	—
Sex	—	—	—	—	—	0.39
Both	8	−1.58 (−1.88, −1.29)	<0.001	95.2	<0.001	—
Male	3	−1.35 (−1.74, −0.97)	<0.001	97.8	<0.001	—
Female	2	−1.01 (−1.96, −0.06)	0.03	99.4	<0.001	—
Duration	—	—	—	—	—	0.10
≤8	9	−1.61 (−1.88, −1.33)	<0.001	96.6	<0.001	—
>8	4	−1.18 (−1.58, −0.78)	<0.001	98.3	<0.001	—
ROB 2	—	—	—	—	—	<0.001
Low	8	−3.44 (−3.82, −3.06)	<0.001	96.2	<0.001	—
Some concerns	5	−0.37 (−0.65, −0.08)	0.01	94.3	<0.001	—
Outcome assessment tool	—	—	—	—	—	<0.001
BDI	10	−1.93 (−2.21, −1.64)	<0.001	97.5	<0.001	—
DASS	2	−0.27 (−0.73, 0.19)	0.25	94.4	<0.001	—
HAM‐D	1	−1.43 (−2.07, 0.78)	<0.001	—	—	—
Anxiety symptom scores						
Health status	—	—	—	—	—	<0.001
Neuropsychiatric disorders	6	−0.78 (−1.12, −0.44)	<0.001	97.1	<0.001	—
Healthy	4	−1.16 (−1.50, −0.81)	<0.001	94.3	<0.001	—
Cancer	2	−5.35 (−6.25, −4.47)	<0.001	98.1	<0.001	—
Sex	—	—	—	—	—	0.012
Both	7	−0.94 (−1.26, −0.62)	<0.001	96.8	<0.001	—
Male	3	−1.67 (−2.04, −1.30)	<0.001	95.4	<0.001	—
Female	2	−1.54 (−2.44, −0.65)	0.001	99.2	<0.001	—
Duration	—	—	—	—	—	<0.001
≤8	8	−1.83 (−2.10, −1.50)	<0.001	96.5	<0.001	—
>8	4	−0.47 (−0.84, −0.10)	0.012	97.8	<0.001	—
ROB 2	—	—	—	—	—	<0.001
Low	8	−2.21 (−2.56, −1.86)	<0.001	97.6	<0.001	—
Some concerns	4	−0.55 (−0.86, −0.24)	<0.001	89.1	<0.001	—
Outcome assessment tool	—	—	—	—	—	<0.001
BAI	8	−2.11 (−2.42, −1.80)	<0.001	97	<0.001	—
DAAS	2	−0.84 (−1.32, −0.36)	0.001	88.2	0.004	—
HAM‐A	2	0.73 (0.19, 1.27)	0.007	97	<0.001	—

*Note*: No, number of trials.

Abbreviations: CI, confidence interval; SMD, standard mean differences.

### 3.6. Grading the Evidence

According to the GRADE framework, the certainty of evidence for the outcome of depressive symptom scores was rated as moderate. This reflects very serious inconsistency, resulting from substantial heterogeneity that persisted even after conducting subgroup analysis, which failed to identify the source of heterogeneity. For this outcome, publication bias was also suspected. In contrast, the outcome of anxiety symptom scores exhibited very serious inconsistency, but the certainty of evidence was rated as high due to an upgrade for the presence of a dose–response gradient (Table S5).

## 4. Discussion

The current meta‐analysis investigated the dose‐dependent effect of crocin on depressive and anxiety symptom scores. Our findings offered that every 30 mg/day increase in crocin supplementation can reduce −3.37 and −2.73 symptom scores associated with depression and anxiety, respectively, suggesting that crocin may have a somewhat greater benefit for depressive symptoms. Nonlinear dose–response analysis also showed declining effects of crocin on depressive and anxiety symptom scores at doses of 30 mg/day (Figures 2 and 4). Regarding the certainty of evidence, the GRADE assessment rated the evidence for anxiety outcomes as high, whereas the evidence for depressive symptom scores was rated as moderate, demonstrating that the clinical evidence for crocin’s anxiolytic effects may be considered somewhat more reliable than that for its antidepressant effects. Although any interpretation requires caution, given the predominance of small single‐country trials. Despite these significant pooled effects, the source of heterogeneity was not revealed by subgroup analysis. Additionally, stratifying the results on the basis of health condition showed larger pooled effect sizes for depressive symptom scores in patient with cancer diseases and then metabolic and neuropsychiatric diseases rather than healthy population. These results were also observed in anxiety, where cancer patients showed a greater reduction in symptom scores than those in healthy participants. This suggests the fact that cancer patients may benefit more from crocin intervention in preventing depression and anxiety disorders. Thereby, these findings indicated pronounced effects of crocin in clinical populations than in healthy individuals. Subgroup analyses further revealed that the BDI detected a significant effect (*p*‐value <0.001), while the DASS did not (*p*‐value = 0.25), indicating that the choice of assessment tool may substantially affect reported outcomes. This discrepancy may be attributed to the different measurement focuses of the instruments. The BDI, as a self‐report measure, primarily captures subjective experiences and cognitive‐affective symptoms, which are often highly sensitive to changes in perceived well‐being. In contrast, while the DASS is a comprehensive tool, its sensitivity to subtle mood shifts or its emphasis on observable and somatic symptoms, as noted in other meta‐analysis [51], might explain why it did not reach statistical significance in this particular context. This observation highlights the necessity for sensitive and standardized outcome measures in future research to ensure consistency across studies.

The results of the current meta‐analysis are aligned with and expand upon prior research, which has indicated that antioxidant compounds, such as crocin, positively influence mental health, particularly by alleviating symptoms of depression and anxiety [52]. In accordance with our findings, previous meta‐analysis by Marx et al. [53] suggested that saffron intervention can reduce the symptoms of depression and anxiety. Marx et al. [53] also highlighted the need for a dose‐finding study to determine the optimal saffron dose and its constituents, noting that most trials have used 30 mg/day. In the present meta‐analysis, we addressed this gap by conducting both linear and nonlinear dose–response analyses [53]. Although the aforementioned study assesses the effect of any form of saffron, in our study, we particularly evaluate the effect of crocin, one of the constituents of saffron herb. While both whole saffron and its bioactive compounds exhibit beneficial effects on health, findings have reported diverse properties across dimensions of well‐being [54]. Thereby, to the best of our knowledge, this is the first systematic review and meta‐analysis to assess the effect of crocin supplementation on depressive and anxiety symptom scores using data from RCTs.

Similarly, in an animal study, crocin had antidepressant and anti‐anxiety‐like activities by reducing inflammation and oxidative stress in the brain and serum corticosterone levels of rats [55]. Another animal study reported that chronic treatment with crocin can significantly reduce depressive‐like behaviors through increasing hippocampus levels of brain‐derived neurotrophic factor (BDNF), CREB, and VGF of rats [56]. A study conducted on depressed rats confirmed that crocin reduced oxidative stress markers and corticosterone levels in the brain, while simultaneously increasing serotonin concentrations. These findings support its role in alleviating depressive‐like and anxiety‐like behaviors [17]. Furthermore, Kazemi et al. [44] reported significantly fewer adverse events in patient in the crocin group compared to fluoxetine group. This suggests that crocin may offer an alternative option for patient seeking effective treatment with potentially reduced side effects. This finding is also consistent with the broader evidence on saffron. A systematic review and meta‐analysis by Yang et al. [57] found that saffron administration did not increase the risk of adverse events compared to either placebo or synthetic antidepressants and concluded that saffron is generally safe. More recently, Shafiee et al. [58] conducted a meta‐analysis comparing saffron against SSRIs, reporting that participants experienced significantly fewer adverse events from saffron than SSRIs, while exhibiting similar efficacy from both treatments for depression and anxiety symptom reduction. However, unlike the synthetic medicine, side effects, including gastrointestinal complications and vertigo, are generally less common with crocin treatment [44, 59]. Common side effects of antidepressants [60]—including sexual dysfunction, weight gain, anticholinergic effects, and arrhythmias—have not been significantly associated with crocin, suggesting that crocin can be comparably more tolerable than conventional medicines. However, it is important to consider the small sample size and short duration of most existing trials. Therefore, long‐term safety of crocin intake, at doses above 30 mg/day, remains uncertain, and calls for more investigation in future studies.

The therapeutic effects of crocin may relate to multiple underlying mechanisms. A primary pathway involves its ability to mitigate oxidative stress and neuroinflammation, both of which are frequently elevated in individuals with depression and anxiety disorders. For instance, crocin inhibits inflammatory pathways, including NF‐kB and NLRP3 signaling, thereby decreasing the production of pro‐inflammatory cytokines, such as TNF‐α and IL‐1β, which are implicated in depression‐ and anxiety‐related behaviors [18]. In humans, meta‐analyses of clinical studies consistently document elevated concentrations of pro‐inflammatory cytokines—IL‐6, TNF‐α, and IL‐1β—in patients with MDD [61, 62]. Beyond these actions, crocin’s antioxidant and anti‐inflammatory properties enhanced neuronal health and resilience, particularly under conditions of chronic stress. Elevated peripheral markers of oxidative damage, like lipid peroxidation products and decreased antioxidant enzyme activity, are documented in MDD patients [63]. Likewise, patients with anxiety disorders display elevated malondialdehyde (MDA), reduced superoxide dismutase (SOD) activity, and dysregulated cytokine profiles [64]. To counteract, crocin can increase the activity of key antioxidant enzymes, such as SOD and catalase (CAT), which protect neural tissue from oxidative damage [19]. Furthermore, crocin elevates the expression of BDNF, a protein essential for neuronal survival, synaptic plasticity, and cognitive function. Higher BDNF levels are associated with improved mood regulation and reduced depression and anxiety, providing an additional basis for crocin’s efficacy [19, 65].

While monoamine hypothesis explained depression as stemming from neurotransmitter deficiency like serotonin, norepinephrine, and dopamine, in vivo and in vitro studies highlight the potential of crocin in antidepressant activity through inhibition of neurotransmitter reuptake [66]. Zhang et al. [67] suggest that crocin I, through its metabolite crocetin, ameliorates anxiety‐like depression symptoms by regulating neurotransmitter reuptake processes. Serotonin is also deficient in anxiety disorder; however, crocin exhibited serotonergic function by inhibiting serotonin reuptake and increasing its availability in synaptic space [68]. Moreover, it acts as a noncompetitive inhibitor of monoamine oxidase‐A (MAO‐A), an enzyme that degrades serotonin, resulting in elevated cerebral serotonin levels [69]. These combined effects indicate that crocin strengthens serotonergic signaling, likely contributing to its anxiolytic and antidepressant properties.

There are some limitations in the present systematic review and meta‐analysis: first, the heterogeneity for outcomes was substantial and remained high even after conducting subgroup analysis. Second, nearly all studies were performed in Iran, and it’s largely attributed to the fact that Iran is the main producer of saffron, from which crocin is derived [70]. While this concentration of research highlights the strong local focus on crocin‐based treatments, it also limits generalizability of our results to other countries. Third, while the overall sample size across the included trials was deemed sufficient to yield a precise pooled estimate in our GRADE assessment (imprecision rated as not serious), there were notable limitations in several individual trials. Particularly, studies by Nishide et al. [40] (whose primary outcome was sleep quality, not depression or anxiety), Talaei et al. [21], and Salek et al. [45] were characterized by small sample sizes, leading to wide CIs for their respective findings. Therefore, our findings should be considered to interpret cautiously. Looking ahead, further investigation is needed to fully elucidate crocin’s potential as a natural, well‐tolerated treatment option for depression and anxiety disorders. Such studies should aim to determine optimal dosing regimens, evaluate long‐term safety and efficacy, and establish uniform assessment protocols to minimize variability. By addressing these priorities, we can more precisely define crocin’s role in depression and anxiety management, leveraging its antioxidant, anti‐inflammatory, and neurochemical attributes to enhance mental health interventions.

Our meta‐analysis possesses several key features. It is the first study to evaluate the net effect of crocin on depressive and anxiety symptom scores using both linear and nonlinear dose–response analysis. Furthermore, all included studies were designed as RCTs, which enhances internal validity by reducing potential source of bias. Most of these trials were also of high quality according to the ROB 2 tool. Additionally, certainty of evidence for depressive and anxiety symptom scores was rated moderate and high, respectively, which made our findings more reliable for clinical recommendations.

## 5. Conclusion

This dose‐dependent meta‐analysis demonstrated that crocin supplementation could have favorable effects on depressive and anxiety symptom scores in adults, with the most pronounced effects around the dose of 30 mg per day. To improve the generalizability of these findings for a broader population, further randomized clinical trials conducted in diverse geographic regions are warranted.

**Nomenclature**
BAI: Beck Anxiety Inventory
BDI: Beck Depression Inventory
BDNF: Brain‐derived neurotrophic factor
CAT: Catalase
CBT: Cognitive behavioral therapy
CI: Confidence interval
CREB: cAMP‐response element binding protein
DAAS: Depression Anxiety and Stress Scale
GRADE: Grading of Recommendations Assessment, Development and Evaluation
MAO‐A: Monoamine oxidase‐A
MDA: Malondialdehyde
MDD: Major depressive disorder
SSRI: Serotonin reuptake inhibitor
GHQ: General Health Questionnaire
HAM‐A: Hamilton Anxiety Rating Scale
HAM‐D: Hamilton Depression Rating Scale
IL‐1β: Interleukin‐1 beta
RCT: Randomized controlled trial
ROB 2: Risk of Bias Tool for Randomized Trials 2
SD: Standard deviation
SMD: Standardized mean difference
SOD: Superoxide dismutase
TNF‐α: Tumor necrosis factor alpha
YLDs: Years lived with disability.

## Author Contributions

Roya Behrouzi and Fatemeh Mehdipoor designed the study, did the literature search, screening data, and performed data extraction. Kimia Torabinasab did the quality assessment and wrote the introduction. Hossein Shahinfar analyzed data and supervised the study. Roya Behrouzi interpreted data and wrote the most part of the manuscript. Fatemeh Mehdipoor wrote methods and some parts of discussion.

## Funding

This research was funded and supported by the Nutritional Health Research Center, School of Health and Nutrition, Lorestan University of Medical Sciences, Khorramabad, Iran (Grant 4462).

## Disclosure

All the authors have read and approved the final manuscript.

## Ethics Statement

The study protocol was approved by the Ethics Committee of Lorestan University of Medical Science (IR.LUMS.REC.1404.223).

## Conflicts of Interest

The authors declare no conflicts of interest.

## Supporting Information

Additional supporting information can be found online in the Supporting Information section.

## Supporting information


**Supporting Information** The following Supporting Information is available through the online version of this article at the publisher’s website. Table S1: PRISMA‐2020 checklist. Figure S1: Flow diagram of the study selection procedure showing the number of eligible randomized controlled trials for the meta‐analysis of the effect of crocin on depressive and anxiety symptom scores. Table S2: Search strategy to find potential eligible studies. Figure S2: Funnel plot displaying publication bias in the studies reporting the impact of crocin on depressive symptoms. Figure S3: Funnel plot displaying publication bias in the studies reporting the impact of crocin on anxiety. Table S3: Excluded studies with reasons for exclusion. Table S4: Risk of bias for randomized controlled trials, assessed according to the revised Cochrane risk‐of‐bias tool for randomized trials (RoB 2). Table S5: Grading the evidence.

## Data Availability

The data that support the findings of this study are available from the corresponding author upon reasonable request.
