# Supplementary material for: Dose‐Dependent Effect of Crocin on Depressive and Anxiety Symptom Scores: A GRADE‐Assessed Systematic Review and Meta‐Analysis of Randomized Controlled Trials
Source: Depress Anxiety. 2026 Oct 30;2026:8303882. doi: 10.1155/da/8303882 (PMC13720669; doi:10.1155/da/8303882)
Supplement: Supplementary file 1 [file DA-2026-8303882-s001.docx]

**Table S1.** PRISMA-2020 checklist.

| **Section and Topic** | **Item #** | **Checklist item** | **Location where item is reported** |
| --- | --- | --- | --- |
| **TITLE** | | |  |
| Title | 1 | Identify the report as a systematic review. | 1 |
| **ABSTRACT** | | |  |
| Abstract | 2 | See the PRISMA 2020 for Abstracts checklist. | 2 |
| **INTRODUCTION** | | |  |
| Rationale | 3 | Describe the rationale for the review in the context of existing knowledge. | 3-4 |
| Objectives | 4 | Provide an explicit statement of the objective(s) or question(s) the review addresses. | 4 |
| **METHODS** | | |  |
| Eligibility criteria | 5 | Specify the inclusion and exclusion criteria for the review and how studies were grouped for the syntheses. | 5 |
| Information sources | 6 | Specify all databases, registers, websites, organisations, reference lists and other sources searched or consulted to identify studies. Specify the date when each source was last searched or consulted. | 4-5 |
| Search strategy | 7 | Present the full search strategies for all databases, registers and websites, including any filters and limits used. | 4, Table S2 |
| Selection process | 8 | Specify the methods used to decide whether a study met the inclusion criteria of the review, including how many reviewers screened each record and each report retrieved, whether they worked independently, and if applicable, details of automation tools used in the process. | 4-5 |
| Data collection process | 9 | Specify the methods used to collect data from reports, including how many reviewers collected data from each report, whether they worked independently, any processes for obtaining or confirming data from study investigators, and if applicable, details of automation tools used in the process. | 5-6 |
| Data items | 10a | List and define all outcomes for which data were sought. Specify whether all results that were compatible with each outcome domain in each study were sought (e.g. for all measures, time points, analyses), and if not, the methods used to decide which results to collect. | 6-7 |
|  | 10b | List and define all other variables for which data were sought (e.g. participant and intervention characteristics, funding sources). Describe any assumptions made about any missing or unclear information. | 4-5 |
| Study risk of bias assessment | 11 | Specify the methods used to assess risk of bias in the included studies, including details of the tool(s) used, how many reviewers assessed each study and whether they worked independently, and if applicable, details of automation tools used in the process. | 5-6 |
| Effect measures | 12 | Specify for each outcome the effect measure(s) (e.g. risk ratio, mean difference) used in the synthesis or presentation of results. | 6-7 |
| Synthesis methods | 13a | Describe the processes used to decide which studies were eligible for each synthesis (e.g. tabulating the study intervention characteristics and comparing against the planned groups for each synthesis (item #5)). | 6-7 |
|  | 13b | Describe any methods required to prepare the data for presentation or synthesis, such as handling of missing summary statistics, or data conversions. | 6 |
|  | 13c | Describe any methods used to tabulate or visually display results of individual studies and syntheses. | 6 |
|  | 13d | Describe any methods used to synthesize results and provide a rationale for the choice(s). If meta-analysis was performed, describe the model(s), method(s) to identify the presence and extent of statistical heterogeneity, and software package(s) used. | 6-7 |
|  | 13e | Describe any methods used to explore possible causes of heterogeneity among study results (e.g. subgroup analysis, meta-regression). | 7 |
|  | 13f | Describe any sensitivity analyses conducted to assess robustness of the synthesized results. | 6-7 |
| Reporting bias assessment | 14 | Describe any methods used to assess risk of bias due to missing results in a synthesis (arising from reporting biases). | 7 |
| Certainty assessment | 15 | Describe any methods used to assess certainty (or confidence) in the body of evidence for an outcome. | 7 |
| **RESULTS** | | |  |
| Study selection | 16a | Describe the results of the search and selection process, from the number of records identified in the search to the number of studies included in the review, ideally using a flow diagram. | 8, Figure S1 |
|  | 16b | Cite studies that might appear to meet the inclusion criteria, but which were excluded, and explain why they were excluded. | 8, Table S3 |
| Study characteristics | 17 | Cite each included study and present its characteristics. | 8, Table 2 |
| Risk of bias in studies | 18 | Present assessments of risk of bias for each included study. | 8, Table 2, Table S4 |
| Results of individual studies | 19 | For all outcomes, present, for each study: (a) summary statistics for each group (where appropriate) and (b) an effect estimate and its precision (e.g. confidence/credible interval), ideally using structured tables or plots. | 8 |
| Results of syntheses | 20a | For each synthesis, briefly summarise the characteristics and risk of bias among contributing studies. | 8-9 |
|  | 20b | Present results of all statistical syntheses conducted. If meta-analysis was done, present for each the summary estimate and its precision (e.g. confidence/credible interval) and measures of statistical heterogeneity. If comparing groups, describe the direction of the effect. | 8-9, Figure 1, Figure 2, Figure 3, Figure 4 |
|  | 20c | Present results of all investigations of possible causes of heterogeneity among study results. | 8-10 |
|  | 20d | Present results of all sensitivity analyses conducted to assess the robustness of the synthesized results. | 8-9 |
| Reporting biases | 21 | Present assessments of risk of bias due to missing results (arising from reporting biases) for each synthesis assessed. | 8-9, Figure S2, Figure S3 |
| Certainty of evidence | 22 | Present assessments of certainty (or confidence) in the body of evidence for each outcome assessed. | 10, Table S5 |
| **DISCUSSION** | | |  |
| Discussion | 23a | Provide a general interpretation of the results in the context of other evidence. | 10 |
|  | 23b | Discuss any limitations of the evidence included in the review. | 13-14 |
|  | 23c | Discuss any limitations of the review processes used. | 13 |
|  | 23d | Discuss implications of the results for practice, policy, and future research. | 14 |
| **OTHER INFORMATION** | | |  |
| Registration and protocol | 24a | Provide registration information for the review, including register name and registration number, or state that the review was not registered. | 2, 4 |
|  | 24b | Indicate where the review protocol can be accessed, or state that a protocol was not prepared. | 4 |
|  | 24c | Describe and explain any amendments to information provided at registration or in the protocol. | 4 |
| Support | 25 | Describe sources of financial or non-financial support for the review, and the role of the funders or sponsors in the review. | 14 |
| Competing interests | 26 | Declare any competing interests of review authors. | 14 |
| Availability of data, code and other materials | 27 | Report which of the following are publicly available and where they can be found: template data collection forms; data extracted from included studies; data used for all analyses; analytic code; any other materials used in the review. | 15 |

*From:*  Page MJ, McKenzie JE, Bossuyt PM, Boutron I, Hoffmann TC, Mulrow CD, et al. The PRISMA 2020 statement: an updated guideline for reporting systematic reviews. BMJ 2021;372:n71. doi: 10.1136/bmj.n71

For more information, visit: <http://www.prisma-statement.org/>

**Identification of studies via databases and registers**

Records removed *before screening*:

Duplicate records removed

(n = 325)

Records identified from:

PubMed (n = 363)

Scopus (n = 687)

Web of Sciences (n = 99)

Manual search (n = 1)

**Identification**

Records screened

(n = 825)

Records excluded

(n = 753)

Reports sought for retrieval

(n = 72)

Reports not retrieved

(n = 0)

**Screening**

Reports excluded:

Non-RCT articles (n = 1)

Other interventions (n=25)

Not measured our outcome (n=29)

Non-English article (n=1)

Congress abstract(n=2)

Full-text articles assessed for eligibility

(n = 72)

Studies included in the systematic review and meta-analysis

(n = 14)

**Included**

**Fig. S1.** Flow diagram of the study selection procedure showing the number of eligible randomized controlled trials for the meta-analysis of the effect of crocin on depression and anxiety symptom scores.

**Table S2.** Search strategy to find potential eligible studies.

| Database | Search strategy | N |
| --- | --- | --- |
| PubMed | ("Crocin"[Title/Abstract] OR "Crocin"[Supplementary Concept] OR "crocus sativus*"[Title/Abstract] OR "crocus"[Title/Abstract] OR "crocus"[MeSH Terms] OR "crocus sativus linn"[Title/Abstract] OR "saffron"[Title/Abstract]) AND ("depressive disorder*"[All Fields] OR "depression"[All Fields] OR "depressive disorder"[MeSH Terms] OR "depression"[MeSH Terms] OR "depress*"[All Fields] OR "depressive symptom*"[All Fields] OR "depressive disorder major"[All Fields] OR "depressive disorder, major"[MeSH Terms] OR "dysthymia"[All Fields] OR "mental health"[All Fields] OR "mental illness"[All Fields] OR "mood"[All Fields] OR "mental disorder*"[All Fields] OR "anxi*"[All Fields] OR "anxiety disorder"[All Fields] OR "anxiety"[All Fields] OR "anxiety"[MeSH Terms] OR "stress*"[All Fields] OR "stress disorder*"[All Fields] OR "affective disorder*"[All Fields] OR "affective illness"[All Fields] OR "psychotic disorder*"[All Fields] OR "psychiatric disorder*"[All Fields] OR "psychiatric illness"[All Fields] OR "HAMD"[All Fields] OR "Hamilton Depression Rating Scale"[All Fields] OR "depression anxiety and stress scale"[All Fields] OR "Beck Depression Inventory"[All Fields]) AND ("Randomized Controlled Trial"[Publication Type] OR "Controlled Clinical Trial"[Publication Type] OR "Clinical Trial"[Publication Type] OR "Random Allocation"[MeSH Terms] OR "Single-Blind Method"[MeSH Terms] OR "Double-Blind Method"[MeSH Terms] OR "Cross-Over Studies"[MeSH Terms] OR "Clinical Trials as Topic"[MeSH Terms] OR "Comparative Study"[Publication Type] OR "Follow-Up Studies"[MeSH Terms] OR "clinical trial*"[Title/Abstract] OR "controlled trial*"[Title/Abstract] OR "intervention"[Title/Abstract] OR "Randomised"[Title/Abstract] OR "Randomized"[Title/Abstract] OR "randomly"[Title/Abstract] OR "placebo"[Title/Abstract] OR "trial"[Text Word] OR "assignment"[Text Word] OR "RCT"[Title/Abstract] OR "cross-over"[Text Word] OR "parallel"[Text Word] OR "single-blind"[Title/Abstract] OR "double-blind"[Title/Abstract] OR "control group"[Title/Abstract]) | **363** |
| Scopus | ( TITLE-ABS-KEY ( crocin ) OR TITLE-ABS-KEY ( "crocus sativus*" ) OR TITLE-ABS-KEY ( safranal ) OR TITLE-ABS-KEY ( saffron* ) ) AND ( TITLE-ABS-KEY ( depress* ) OR TITLE-ABS-KEY ( dysthymia ) OR TITLE-ABS-KEY ( "mental health" ) OR TITLE-ABS-KEY ( "mental illness" ) OR TITLE-ABS-KEY ( "mental disorder*" ) OR TITLE-ABS-KEY ( anxi* ) OR TITLE-ABS-KEY ( stress* ) OR TITLE-ABS-KEY ( psychotic* ) ) AND ( TITLE-ABS-KEY ( "Randomized Controlled Trial" ) OR TITLE-ABS-KEY ( "Controlled Clinical Trial" ) OR TITLE-ABS-KEY ( "Clinical Trial" ) OR TITLE-ABS-KEY ( "Random Allocation" ) OR TITLE-ABS-KEY ( "Single-Blind Method" ) OR TITLE-ABS-KEY ( "Double-Blind Method" ) OR TITLE-ABS-KEY ( "Cross-Over Studies" ) OR TITLE-ABS-KEY ( "Comparative Study" ) OR TITLE-ABS-KEY ( "Follow-Up Studies" ) OR TITLE-ABS-KEY ( "trial*" ) OR TITLE-ABS-KEY ( intervention* ) OR TITLE-ABS-KEY ( random* ) OR TITLE-ABS-KEY ( placebo ) OR TITLE-ABS-KEY ( rct ) | **687** |
| ISI Web of Science | **Crocin OR “crocus sativus*” OR “crocus sativus linn” OR saffranal OR saffron (Topic) and "depressive disorder*” OR "depression" OR "depress*" OR "depressive symptom*” OR "depressive disorder, major" OR “dysthymia” OR "mental health" OR "mental illness" OR "mood" OR "mental disorder*" OR “anxi*” OR "anxiety disorder" OR "anxiety" OR "stress*" OR "stress disorder*" OR “affective disorder*” OR “affective illness” OR "psychotic disorder*" OR “psychiatric disorder*” OR “psychiatric illness” OR "hand" OR "Hamilton Depression Rating Scale" OR "depression anxiety and stress scale" OR "Beck Depression Inventory" (All Fields) and "Randomized Controlled Trial" OR "Controlled Clinical Trial" OR "Clinical Trial" OR "Random Allocation" OR "Single-Blind Method" OR "Double-Blind Method" OR "Cross-Over Studies" OR "Clinical Trial" OR "Comparative Study" OR "Follow-Up Studies" OR "clinical trial*" OR "controlled trial*" OR "intervention*" OR "Randomised" OR "Randomized" OR "Pilot study" OR "randomly" OR "placebo" OR "trial" OR "assignment" OR "RCT" OR "cross-over" OR "parallel" OR "single-blind" OR "double-blind" OR “control group”** | **99** |
| All | PubMed + Scopus + ISI Web of Science | **1149** |

**Table S3**. Excluded studies with reasons for exclusion

| No. | Title | First author | Exclusion reason |
| --- | --- | --- | --- |
| 1 | Hydro-alcoholic extract of Crocus sativus L. versus fluoxetine in the  treatment of mild to moderate depression: a double-blind,  randomized pilot trial | Noorbala, A.A. | other saffron constituents (stigma of saffron) |
| 2 | Crocus sativus L. in the Treatment of Mild to Moderate Depression: A Double-blind, Randomized and Placebo-controlled Trial | Akhondzadeh, S. | other saffron constituents (stigma of saffron) |
| 3 | اثر زعفران ( (Crocus sativus Lدر درمان افسردگي خفيف تا متوسط: يك كارآزمايي باليني تصادفي به شيوه دوسوبيخبر و با كنترل دارونما | Noorbala, A.A. | other saffron constituents (stigma of saffron) |
| 4 | Evaluation of Crocus sativus L. (saffron) on male erectile dysfunction:  A pilot study | Shamsa, A. | other saffron constituents (stigma of saffron) |
| 5 | Psychological and neuroendocrinological effects of saffron scent: Crossover study with 35 young female test persons | Schulz, V. | Non-English article |
| 6 | Saffron (crocus sativus) in the treatment of anxiety and depression: a double-blind, randomized and placebo-controlled trial | Shemshian, M. | Poster article |
| 7 | Effect of saffron on fluoxetine-induced sexual impairment in men: randomized double-blind placebo-controlled trial | Modabernia, A. | Not pure crocin (stigma + crocin) |
| 8 | Safety evaluation of crocin (a constituent of saffron) tablets in healthy volunteers | Mohamadpour, A. | Incorrect outcome |
| 9 | Saffron aqueous extract prevents metabolic syndrome in patients with schizophrenia on olanzapine treatment: A randomized triple blind placebo controlled study | Fadai, F. | Incorrect outcome |
| 10 | Effect of Crocin on Major Depressive Disorder: A Double Blind Clinical Trial | Mohajeri, S. | Meeting absteract |
| 11 | Comparing the efficacy and safety of Crocus sativus L. with memantine in patients with moderate to severe Alzheimer's disease: A double-blind randomized clinical trial | Farokhnia, M. | Incorrect outcome |
| 12 | A randomized, double-blind, clinical trial comparing the efficacy and safety of Crocus sativus L. with fluoxetine for improving mild to moderate depression in post percutaneous coronary intervention patients | Shahmansouri | Not pure crocin (whole saffron extract) |
| 13 | Safety evaluation of saffron stigma Crocus sativus L.) aqueous extract and crocin in patients with schizophrenia | Mousavi, B. | Incorrect outcome |
| 14 | Study the effects of saffron on depression and lipid profiles: A double blind comparative study | Sahraian , A. | Not pure crocin (whole saffron extract) |
| 15 | Efficacy and safety of crocus sativus L. in patients with mild cognitive impairment: One year single-blind randomized, with parallel groups, clinical trial | Tsolakib, M. | Not pure crocin (whole saffron extract) |
| 16 | Saffron and crocin improved appetite, dietary intakes and body composition in patients with coronary artery disease | Abedimanesh, N. | Incorrect outcome |
| 17 | Crocus sativus L. versus Citalopram in the Treatment of Major Depressive Disorder with Anxious Distress: A Double-Blind, Controlled Clinical Trial | Ghajar, A. | Not pure crocin (whole saffron) |
| 18 | Effect of Saffron (Crocus sativus L.) as an Add-On Therapy to Sertraline in Mild to Moderate Generalized Anxiety Disorder: A Double Blind Randomized Controlled Trial | Jafarnia, N | Not pure crocin (whole saffron exteract) |
| 19 | Comparison of Saffron versus Fluoxetine in Treatment of Mild to Moderate Postpartum Depression: A Double-Blind, Randomized Clinical Trial | Kashani, L | Not pure crocin (whole saffron exteract) |
| 20 | The Effect of Saffron on Depression among Recovered Consumers of Methamphetamine Living with HIV/AIDS | Jalali, F. | Not pure crocin (whole saffron exteract) |
| 21 | Saffron improved depression and reduced homocysteine level in patients with major depression: A Randomized, double-blind study | Jelodar, G. | Not pure crocin (whole saffron exteract) |
| 22 | Effects of Crocin on Diabetic Maculopathy: A Placebo-Controlled Randomized Clinical Trial | Sepahi, S. | Incorrect outcome |
| 23 | Effects of crocin in reducing DNA damage, inflammation, and oxidative stress in multiple sclerosis patients: A double-blind, randomized, and placebo-controlled trial | Ghiasiani, M. | Incorrect outcome |
| 24 | Therapeutic effects of saffron (Crocus sativus) versus fluoxetine on Irritable Bowel Syndrome: A double-blind randomized clinical trial | Najafabadi, B. | Not pure crocin (whole saffron exteract) |
| 25 | Effects of crocin and saffron aqueous extract on gene expression of SIRT1, AMPK, LOX1, NF-κB, and MCP-1 in patients with coronary artery disease: A randomized placebo-controlled clinical trial | Abedimanesh, N. | Incorrect outcome |
| 26 | Probable antioxidant therapy of saffron crocin in patients with multiple sclerosis: A randomized controlled trial | Ahmadi, S.A. | Incorrect outcome |
| 27 | The effect of crocin supplementation on glycemic control, insulin resistance and active AMPK levels in patients with type 2 diabetes: A pilot study | Behrouz, V. | Incorrect outcome |
| 28 | Effects of Saffron Extract Supplementation on Mood, Well-Being, and Response to a Psychosocial Stressor in Healthy Adults: A Randomized, Double-Blind, Parallel Group, Clinical Trial | Jackson | Not pure crocin (whole saffron exteract) |
| 29 | Saffron for the management of premenstrual dysphoric disorder: A randomized controlled trial | Rajabi, F. | Not pure crocin (whole saffron exteract) |
| 30 | The effect of crocin (the main active saffron constituent) on the cognitive functions, craving, and withdrawal syndrome in opioid patients under methadone maintenance treatment | Abbaszadeh-Mashkani | Incorrect outcome |
| 31 | Impact of saffron (Crocus Sativus Linn) supplementation and resistance training on markers implicated in depression and happiness levels in untrained young males | Moghadam, B | Not pure crocin (whole saffron exteract) |
| 32 | Effects of Saffron Extract on Sleep Quality: A Randomized Double-Blind Controlled Clinical Trial | Pachikian, B.D. | Incorrect outcome |
| 33 | Comparing the effects of saffron, lippia, and saffron-lippia combination on anxiety among candidates for coronary angiography | Soheilipur, K | Not pure crocin (whole saffron exteract) |
| 34 | Cardioprotective effect of saffron total glycoside tablets in patients with breast cancer receiving anthracycline-based chemotherapy: Study protocol for a multicentre, randomised, parallel, double-blind, placebo-controlled clinical trial | Su, X | Study protocol |
| 35 | Saffron improves life and sleep quality, glycaemic status, lipid profile and liver function in diabetic patients: A double-blind, placebo-controlled, randomised clinical trial | Tajaddini | Not pure crocin (whole saffron exteract) |
| 36 | Effect of Crocin From Saffron (Crocus sativus L.) Supplementation on Oxidant/Antioxidant Markers, Exercise Capacity, and Pulmonary Function Tests in COPD Patients: A Randomized, Double-Blind, Placebo-Controlled Trial | Ghobadi, H. | Incorrect outcome |
| 37 | Evaluation of therapeutic effects of crocin in attenuating the progression of diabetic nephropathy: a preliminary randomized triple-blind placebo-controlled trial | Jaafarinia. A | Incorrect outcome |
| 38 | Effect of crocin on diabetic patients: A placebo-controlled, triple-blinded clinical trial | Sepahi, S | Incorrect outcome |
| 39 | Efficacy and safety of saffron as adjunctive therapy in adults with attention-deficit/hyperactivity disorder: A randomized, double-blind, placebo-controlled clinical trial | Pazoki, B. | Incorrect outcome |
| 40 | Evaluation of the effect of crocin on prevention of atrial fibrillation after coronary artery bypass grafting or heart valve replacement: A double-blind, randomized, placebo-controlled trial | Shahri, S. | Incorrect outcome |
| 41 | Evaluation of Crocin Effect on Contrast-Induced Nephropathy Following Coronary Angiography or Angioplasty: A Randomized Controlled Trial | Shojaei, L. | Incorrect outcome |
| 42 | Evaluation of Saffron (Crocus Sativus L) Effects on Anxiety in Hospitalized Patients with Acute Coronary Syndrome | Askari, F | other saffron constituents (stigma of saffron) |
| 43 | Effect of crocin of Crocus sativus L. on serum inflammatory markers (IL-6 and TNF-α) in chronic obstructive pulmonary disease patients: a randomised, double-blind, placebo-controlled trial | Aslani, M. | Incorrect outcome |
| 44 | The effect of saffron and corrective exercises on depression and quality of life in women with multiple sclerosis: A randomized controlled clinical trial | Bahrami, F. | Not pure crocin (whole saffron exteract) |
| 45 | Acute Effect of a Saffron Extract (Safr'Inside(TM)) and Its Main Volatile Compound on the Stress Response in Healthy Young Men: A Randomized, Double Blind, Placebo-Controlled, Crossover Study | Pouchieu, C. | Not pure crocin (whole saffron exteract) |
| 46 | Effect of Crocus sativus Extract Supplementation in the Metabolic Control of People with Diabetes Mellitus Type 1: A Double-Blind Randomized Placebo-Controlled Trial | Giannoulaki, P. | Not pure crocin (whole saffron exteract) |
| 47 | Crocin supplementation in primary open angle glaucoma: A randomized, triple-blind, placebo-controlled clinical trial | Mahdiani, S. | Incorrect outcome |
| 48 | Effects of crocin and saffron aqueous extract on gene expression of SIRT1, AMPK, LOX1, NF-κB, and MCP-1 in patients with coronary artery disease: A randomized placebo-controlled clinical trial | Abedimanesh, N. | Incorrect outcome |
| 49 | Crocus Sativus L. (saffron) versus sertraline on symptoms of depression among older people with major depressive disorders-a double-blind, randomized intervention study | Ahmadpanah, M. | Not pure crocin (whole saffron exteract) |
| 50 | Inflammatory markers response to crocin supplementation in patients with type 2 diabetes mellitus: A randomized controlled trial | Behrouz, V. | Incorrect outcome |
| 51 | Effect of crocin, a carotenoid from saffron, on plasma cholesteryl ester transfer protein and lipid profile in subjects with metabolic syndrome: A double blind randomized clinical trial | Javandoost, A. | Incorrect outcome |
| 52 | Saffron for treatment of fluoxetine-induced sexual dysfunction in women: randomized double-blind placebo-controlled study | Kashani, L. | Not pure crocin (whole saffron exteract) |
| 53 | The effect of crocin on movement disorders and oxidative DNA damage in Parkinson's disease: Insights from a randomized controlled trial | Soleymani, S. M. | Incorrect outcome |
| 54 | The effects of crocus sativus extract on serum lipid profile and liver enzymes in patients with non-alcoholic fatty liver disease: A randomized placebo-controlled study | Parsi, A. | Incorrect outcome |
| 55 | Saffron (crocus sativus) in the treatment of anxiety and depression: a double-blind, randomized and placebo-controlled trial | Shemshian, M. | Not pure crocin (whole saffron exteract) |
| 56 | A pilot study of the effects of crocin on high-density lipoprotein cholesterol uptake capacity in patients with metabolic syndrome: A randomized clinical trial | Saberi-Karimian, M. | Incorrect outcome |
| 57 | Effectiveness of crocin of saffron (Crocus sativus L.) against chemotherapy-induced peripheral neuropathy: A randomized, double-blind, placebo-controlled clinical trial | Bozorgi, H. | Incorrect outcome |
| 58 | The Effects of Crocin Supplementation on Sleep Quality, General Health, and Happiness in Elderly Population: A Randomized, Double-blind, Placebo-controlled Trial | Sadeghi-Gandomani, H. | Incorrect outcome |

**Table S4**. Risk of bias for randomized controlled trials, assessed according to the Revised Cochrane risk-of-bias tool for randomized trials (RoB 2).

| **Author, year** | **Randomization process** | **Deviations from the intended interventions** | **Missing outcome data** | **Measurement of the outcome** | **Selective reporting** | **Overall assessment of risk if bias** |
| --- | --- | --- | --- | --- | --- | --- |
| Abedimanesh, 2017 | L | L | L | L | L | L |
| Ebrahimi, 2024 | L | L | L | L | L | L |
| Ghaderi, 2019 | L | L | L | L | L | L |
| Nikbakht Jam, 2017 | S | L | L | L | L | S |
| Jomehpour, 2022 | L | S | L | L | L | S |
| Kazemi, 2020 | L | L | L | L | L | L |
| Khalatbari-mohseni, 2019 | L | L | L | L | L | L |
| Kouchaki, 2024 | L | L | S | L | L | S |
| Kolahdooz, 2023 | S | S | L | L | L | S |
| Mahdavi, 2024 | L | L | L | L | L | L |
| Nishide, 2018 | L | L | L | L | L | L |
| Pakfetrat, 2019 | S | L | L | L | L | S |
| Sadeghi-Gandomani, 2023 | L | L | L | L | L | L |
| Salek, 2021 | L | L | L | L | L | L |
| Talaei, 2015 | L | L | L | L | L | L |

**Fig S2**: Funnel plot displaying publication bias in the studies reporting the impact of crocin on depressive symptom scores

**Figure S3**: Funnel plot displaying publication bias in the studies reporting the impact of crocin on anxiety symptom scores

**Table S5:** Grading the evidence

| **Certainty assessment** | | | | | | | **№ of patients** | | **Effect** | | **Certainty** | **Importance** |
| --- | --- | --- | --- | --- | --- | --- | --- | --- | --- | --- | --- | --- |
| **№ of studies** | **Study design** | **Risk of bias** | **Inconsistency** | **Indirectness** | **Imprecision** | **Other considerations** | **Crocin** | **Control** | **Relative (95% CI)** | **Absolute (95% CI)** |  |  |
| **Depressive symptom scores (follow-up: range 4 weeks to 16 weeks; assessed with: Questionnaire)** | | | | | | | | | | | | |
| 12 | randomised trials | not serious | very serious^a^ | not serious | not serious | publication bias strongly suspected all plausible residual confounding would reduce the demonstrated effect dose response gradient^b^ | 281 | 224 | - | MD **3.11 lower** (4.75 higher to 1.65 higher) | ⨁⨁⨁◯ Moderate^a,b^ | IMPORTANT |
| **Anxiety Symptom scores (follow-up: range 4 weeks to 16 weeks; assessed with: Questionnaire)** | | | | | | | | | | | | |
| 12 | randomised trials | not serious | very serious^a^ | not serious | not serious | all plausible residual confounding would reduce the demonstrated effect dose response gradient | 307 | 300 | - | MD **2.55 lower** (4.04 higher to 1.06 higher) | ⨁⨁⨁⨁ High^a^ | IMPORTANT |

**CI:** confidence interval; **MD:** mean difference

**Explanations**

a. Heterogeneity among studies was high (I2 > 75%).

b. Publication bias was found according to funnel plot, Egger and Begg Tests.
